# Cardiac Phase-Resolved T_2_^*^ Magnetic Resonance Imaging Reveals Differences Between Normal Hearts and a Humanized Mouse Model of Hypertrophic Cardiomyopathy

**DOI:** 10.3390/biomedicines13051193

**Published:** 2025-05-14

**Authors:** Oumaima Laghzali, Shahriar Shalikar, Siqin Liu, Sandra Lehmann, Joao dos Santos Periquito, Andreas Pohlmann, Sonia Waiczies, Lucie Carrier, Hsin-Jung Yang, Thoralf Niendorf, Min-Chi Ku

**Affiliations:** 1 Max-Delbrück-Center for Molecular Medicine in the Helmholtz Association (MDC), Berlin Ultrahigh Field Facility (B.U.F.F.), 13092 Belin, Germany; oumaima.laghzali@mdc-berlin.de (O.L.); shahriar.shalikar@mdc-berlin.de (S.S.); siqin.liu@mdc-berlin.de (S.L.); sandra_lehmann95@web.de (S.L.); joao.s.periquito@gmail.com (J.d.S.P.); a.pohlmann.phd@gmail.com (A.P.); sonia.waiczies@mdc-berlin.de (S.W.); thoralf.niendorf@mdc-berlin.de (T.N.); 2 Charité—Universitätsmedizin Berlin, Corporate Member of Freie Universität Berlin and Humboldt Universität zu Berlin, 10117 Berlin, Germany; 3 DZHK (German Centre for Cardiovascular Research), Partner Site Berlin, 10115 Berlin, Germany; 4 Experimental and Clinical Research Center (ECRC), a Joint Cooperation between the Charité Medical Faculty and the Max-Delbrück Center for Molecular Medicine, 13125 Berlin, Germany; 5 Department of Experimental Pharmacology and Toxicology, University Medical Center Hamburg-Eppendorf, 20246 Hamburg, Germany; l.carrier@uke.de; 6 DZHK (German Centre for Cardiovascular Research), Partner Site Hamburg/Kiel, Lübeck, 20246 Hamburg, Germany; 7 Biomedical Imaging Research Institute, Cedars-Sinai Medical Center, Los Angeles, CA 90048, USA; hsin-jung.yang@cshs.org

**Keywords:** hypertrophic cardiomyopathy (HCM), myocardial tissue characterization, cardiac MRI, parametric imaging, T_2_^*^ mapping

## Abstract

**Background/Objectives**: While T_2_^*^ mapping effectively assesses cerebral blood oxygenation, its utility for capturing cardiac phase-dependent myocardial changes in hypertrophic cardiomyopathy (HCM) is underexplored. This study investigates T_2_^*^ dynamics in an HCM mouse model, to validate T_2_^*^ as a clinically relevant biomarker for improved HCM diagnosis and treatment monitoring. **Methods**: A cardiac-specific *Mybpc3* genetic mouse model, closely mirroring human HCM, was used with 12 young mice (6–11 weeks old), including both male and female wild-type (WT) and *Mybpc3*-KI (HCM) groups. The cardiac function was assessed using self-gated multi-slice 2D CINE imaging. To investigate myocardial T_2_^*^ variations across the cardiac cycle, multi-gradient echo (MGE) imaging was employed. This approach used retrospective gating and continuous acquisition synchronization with pulse oximetry at 9.4 T small animal MRI. **Results**: *Mybpc3*-KI mice demonstrated left-ventricular (LV) hypertrophy compared to WT (HCM = 50.08 ± 4.68 µm/g vs. WT = 45.80 ± 20.07 µm/g, *p* < 0.01) and reduced ejection fraction (HCM = 38.55 ± 5.39% vs. WT= 72.53 ± 3.95%, *p* < 0.01). Myocardial T_2_^*^ was significantly elevated in HCM across all cardiac phases (HCM = 12.14 ± 1.54 ms vs. WT = 7.93 ± 1.57 ms, *p* = 0.002). Strong correlations were observed between myocardial T_2_^*^ and LV mass (rho = 0.88, *p* = 0.03). **Conclusions**: T_2_^*^ was elevated in HCM with increased LV mass, highlighting the potential of T_2_^*^ MRI as a sensitive biomarker for distinguishing healthy mice from those with HCM and revealing possible myocardial abnormalities.

## 1. Introduction

Hypertrophic cardiomyopathy (HCM) is a genetically driven cardiac disorder with a prevalence of 200 to 500 cases per 100,000 individuals [1,2]. It is most commonly caused by mutations in genes encoding sarcomeric proteins, particularly *MYH7* (OMIM #115197) and *MYBPC3* (OMIM #600958), yet its clinical presentation can differ widely in both age of onset and severity [3,4,5]. One of the major challenges in managing HCM lies in the early detection of myocardial alterations, such as myocardial fibrosis and microvascular dysfunction, that precede overt structural changes and may dynamically fluctuate throughout the cardiac cycle [6,7,8,9,10,11].

Magnetic resonance imaging (MRI) provides the means for myocardial tissue assessment in HCM. Current MRI approaches rely on covering a single cardiac phase, most often diastole [12]. While this strategy captures important structural information, it provides only a static snapshot of a highly dynamic organ. Key physiological properties, such as myocardial oxygenation, perfusion, and mechanical strain, vary significantly between systole and diastole [6,7,8], and these temporal fluctuations are likely to hold diagnostic relevance, particularly in the early stages of HCM when overt left ventricular hypertrophy (LVH) is absent.

Earlier studies have leveraged quantitative cardiac magnetic resonance (CMR) techniques, such as quantitative mapping of the MRI metric transversal effective relaxation time (T_2_^*^), to investigate these changes because of its sensitivity to myocardial oxygenation, perfusion, and fibrotic remodeling [13,14,15,16,17,18]. At ultrahigh magnetic field strengths, T_2_^*^ values have been shown to correlate with myocardial wall thickness in HCM, suggesting increased sensitivity to early tissue abnormalities [19,20]. Ultrahigh field MRI enhances the signal-to-noise ratio and susceptibility contrast, enabling more precise detection of subtle myocardial changes that may be undetectable at lower magnetic field strengths [21,22]. However, implementing this approach in human studies remains technically challenging. Magnetohydrodynamic (MHD) effects distort the electrocardiogram (ECG) signal at high magnetic field strengths, which presents a challenge for the synchronization of data acquisition with cardiac activity [23,24,25]. These issues are particularly pronounced in small animal models due to their high heart rates [26].

To overcome these challenges and to capture myocardial T_2_^*^ fluctuations across the cardiac cycle, we developed a retrospectively gated, cardiac phase-resolved T_2_^*^ mapping technique that synchronizes image acquisition with the cardiac cycle, enabling the dynamic assessment of myocardial tissue properties [27,28]. To investigate the underlying mechanisms in a controlled setting, we back-translated clinical observations into a humanized HCM mouse model at a defined pathological stage. This approach captures dynamic changes that may be missed in conventional single-phase T_2_^*^ mapping. By providing mechanistic insights into myocardial tissue alterations, this approach has the potential to refine clinical imaging strategies for earlier and more precise HCM detection, potentially improving risk stratification and patient management.

## 2. Materials and Methods

### 2.1. Mouse Model Carrying Human HCM Gene Mutation

All animal studies were designed and handled in accordance with ARRIVE guidelines, with EU Directive 2010/63/EU and Regulation (EU) 2019/1010, and were approved by the local state review board with ethical approval number G0257/18. The *Mybpc3*-KI HCM mouse model, carrying a heterozygous G > A point mutation on the last nucleotide of exon 6 in the myosin binding protein C3 (*Mybpc3*) gene on a C57BL/6J background [29], was bred and maintained in the institutional animal facility to generate homozygous *Mybpc3*-KI (KI) mice and their wild-type (WT) littermate controls. Both sexes were included to account for potential sex-based differences in disease progression. In total, 3 female and 3 male WT mice, along with 3 female and 3 male KI mice aged 6–11 weeks old were used for in vivo cardiac MRI.

### 2.2. Free Breathing, Cardiac Phased-Resolved T_2_^*^ Mapping

**Figure 1:**
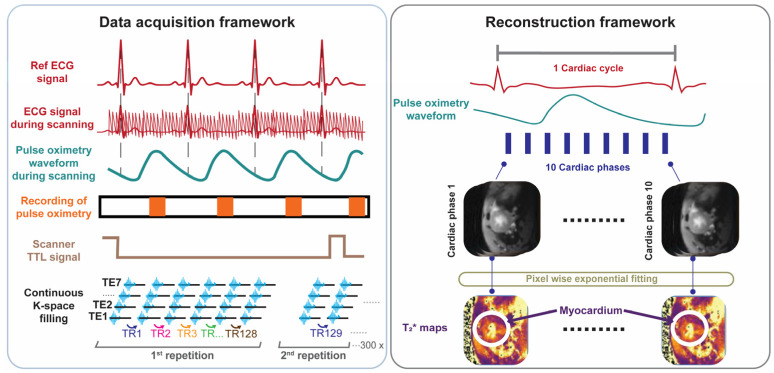
Data acquisition and retrospective gated image reconstruction scheme used for cardiac phase-resolved, whole cardiac cycle T_2_^*^ mapping of the mouse heart. Data acquisition framework: MRI data acquisition is performed continuously. Synchronization is achieved by recording the pulse-oximetry trace and the scanner’s TTL trigger signal. Following data collection, retrospective gating based on recorded signals is performed. Reconstruction framework: Each k-space line is assigned to the corresponding cardiac phase and then averaged to yield 10 cardiac phases, each with 7 echoes obtained for different echo times. T_2_^*^ maps are generated via pixel-wise mono-exponential fitting of the T_2_^*^ signal decay.

For probing T_2_^*^ across the cardiac cycle we developed, implemented, and validated free breathing, retrospectively gated cardiac phase-resolved T_2_^*^ mapping with full R-R interval coverage. This approach involves continuous acquisition of multi-gradient-echo (MGE) data during cardiac motion (Figure 1). Reconstruction of the cardiac phase-resolved images is based on the timing of the cardiac events. This technique relies on sorting raw data into cardiac phases using external cardiac triggering signals, such as those derived from pulse oximetry or simulated heartbeats. Retrospective gating was used to enable image reconstruction for each phase of the cardiac cycle and to facilitate probing myocardial T_2_^*^ dynamics across all cardiac phases.

To validate the retrospectively gated cardiac phase-resolved T_2_^*^ mapping approach, we performed a phantom study using simulated heart beats for reconstruction. An NMR tube phantom containing six different iron concentrations (0.75–5 µL ferumoxytol/mL distilled water, ferumoxytol stock solution (0.03 g/mL iron) was prepared to mimic myocardial T_2_^*^ properties (T_2_^*^ = 4–20 ms) at 9.4T. The phantom was scanned using a fully sampled 2D multi-gradient-echo (MGE) sequence with 300 continuous measurements and linear sequential Cartesian phase-encoding. To test the robustness of the reconstruction method, artificial heartbeat patterns mimicking the typical mouse heart rate range were emulated. For validation, various conditions were examined, including different repetition numbers, cardiac phase timing variations, and variable cardiac cycle durations typical of mouse imaging conditions. Second-order B_0_ shimming was applied to minimize magnetic field inhomogeneity while image quality was assessed using signal-to-noise ratio (SNR) measurements following the National Electrical Manufacturers Association (NEMA) single-image method [30]. Two reconstruction approaches were compared: a conventional MGE reconstruction (reference method) and a retrospective binning method that sorted data based on the simulated cardiac timing and the scanner’s binary Transistor–Transistor Logic (TTL) output signal (detailed parameters of the MRI protocol are provided in Table 1).

### 2.3. In Vivo Cardiac MRI

In vivo cardiac MRI was conducted on a 9.4T small bore animal MRI system (BioSpec 94/20, Bruker BioSpin, Ettlingen, Germany) using a 72 mm linear volume resonator for transmission and a 4-channel surface cardiac RF array for signal reception (Bruker BioSpin MRI GmbH, Ettlingen, Germany). The mice were anesthetized using 3% isoflurane (CP-Pharma, Burgdorf, Germany) in 300 mL/min medical air and 250 mL/min oxygen, and maintained at 1 to 1.5% isoflurane after induction throughout the imaging. Their heart rate, respiration, and core body temperature were monitored using a gating system (Model 1030, SA Instruments Inc., New York, NY, USA). Two-dimensional CINE images of the whole heart were acquired for cardiac chamber quantification and for cardiac function assessment. For this purpose, 7–8 short axis (SAX) slices covering the whole heart were consecutively acquired using self-gated bright-blood CINE IntraGate-FLASH with scanning parameters provided in Table 1.

### 2.4. Cardiac Phase-Resolved T_2_^*^ Mapping

Following phantom validation, the retrospectively gated cardiac phase-resolved T_2_^*^ mapping method was implemented for in vivo mouse imaging covering a mid-ventricular slice of a short axis view (SAX) of the heart. Before MGE data acquisition, and to minimize off-resonance artifacts, the 2nd order shimming ROI was positioned as tightly as possible around the myocardium, ensuring minimal artifact interference in critical regions near the air–tissue interface. During data acquisition, MGE raw data were continuously acquired using sequential linear phase encoding (Figure 1). Simultaneously, a cardiac triggering signal was recorded using fiber optic pulse oximetry (Model 1030, SA Instruments Inc., New York, NY, USA) along with a TTL output signal generated by the pulse sequence, indicating the start and the end of each whole k-space measurement. Both signals were simultaneously recorded and digitized using an analog-to-digital converter (DT 9800-16SE-BNC, Data Translation GmbH, Bietigheim-Bissinge, Germany) and exported through simplified data acquisition software (HSE-Haemodyn, Version 1.5, Hugo Sachs Elektronik–Harvard Apparatus GmbH, March-Hugstetten, Germany).

Each k-space readout obtained for all of the echoes was binned into the corresponding cardiac phases based on MRI data acquisition timestamps, obtained from the TTL recordings, and pulse oximetry peaks. R-R intervals deviating by more than 5% from the moving median of 10 consecutive heartbeats were excluded to prevent errors caused by pulse-oximetry trigger failures, which sometimes resulted in incorrect cardiac cycle durations. This method, adapted from Di Sopra et al. [31], helped remove outliers while capturing natural variations in the heart rate.

Based on the validation study in phantoms, the number of cardiac phases of 10 was set to reconstruct the cardiac phase-resolved multi-gradient-echo images followed by T_2_^*^ quantification, as outlined in the reconstruction framework shown in Figure 1. A total of 10 cardiac phases were chosen to balance the competing constraints of temporal resolution, image quality, heart rate, and hardware limitations to ensure reliable T_2_^*^ quantification while maintaining a feasible scan time. By using a short TR (~100 ms) and carefully optimized TE inter-echo spacing across multiple echoes, we achieved sufficient temporal resolution to capture the key cardiac phases—especially end-systole—within a ~10-min scan. This configuration allowed for accurate T_2_^*^ curve fitting while staying within the duty cycle limits of the gradient system, making it well-suited for retrospective cardiac–respiratory gated small animal MRI.

The allocated k-space lines for each phase and gradient-echo were processed with a reconstruction pipeline, including zero- and first-order phase correction, complex averaging, and fast Fourier transform (FFT) (Figure 1). The multi-gradient-echo data obtained for each cardiac phase were used for pixelwise mono-exponential fitting to generate a T_2_^*^ map for each distinct cardiac phase (Figure 1). A cardiac phase shift correction was applied to ensure that the first phase aligns with the onset of systole.

Data sorting, image reconstruction, and T_2_^*^ quantification were performed offline in a workstation using custom-developed MATLAB (R2021a; MathWorks, Inc., Natick, MA, USA) scripts.

### 2.5. Data Analysis

Cardiac chamber quantification and cardiac function assessment were performed using manual segmentation based on the AHA 17-segment model in the open-source software Segment v4.0 R11044b (Medviso, segment.heiberg.se, Lund, Sweden) [32]. Endo- and epicardial borders were manually segmented in end-systole and end-diastole using a stack of SAX views derived from CINE imaging. From these segmentations, left ventricular (LV) ejection fraction (EF), LV myocardial mass, and LV wall thickness were calculated. End-diastolic volume (EDV) and end-systolic volume (ESV) were derived to quantify the volumetric status of the LV at maximum filling and maximum contraction.

For T_2_^*^ assessment, a ROI covering the myocardium of the left ventricle was defined. The mask was manually drawn using a home-built segmentation code in MATLAB. The T_2_^*^ of all pixels within the myocardial ROI was averaged for further analysis.

### 2.6. Statistical Analysis

Experimental statistics were conducted, and graphs were generated in R studio version 4.4.1 (R Studio Inc., Boston, MA, USA; http://www.rstudio.com/) and MATLAB.

For the phantom study, T_2_^*^ measurements were analyzed using both the retrospective gating method and the reference method, and their agreement was assessed using linear regression and Pearson’s R^2^. Intraclass correlation coefficients (ICCs) were determined to examine the agreement between both methods. For the animal study (*n* = 12), non-parametric tests were used: Mann–Whitney U for group comparisons, Spearman correlation for relationships, and the Friedman test for T_2_^*^ differences across the cardiac phases. The Friedman test statistic (χ^2^: Chi-squared) quantifies the magnitude of differences across repeated measurements within groups. When the Friedman test indicated significant differences, post hoc pairwise comparisons were performed using Wilcoxon signed-rank tests with Holm’s correction. For the Spearman correlation, rho (ρ) was used as the correlation coefficient, with correlation strength defined as follows: very weak (0.00–0.19), weak (0.20–0.39), moderate (0.40–0.59), strong (0.60–0.79), and very strong (0.80–1.00). Differences were considered statistically significant with * *p* < 0.05 with ** *p* < 0.01, *** *p* < 0.001.

## 3. Results

### 3.1. Validation in Phantom Study

**Figure 2:**
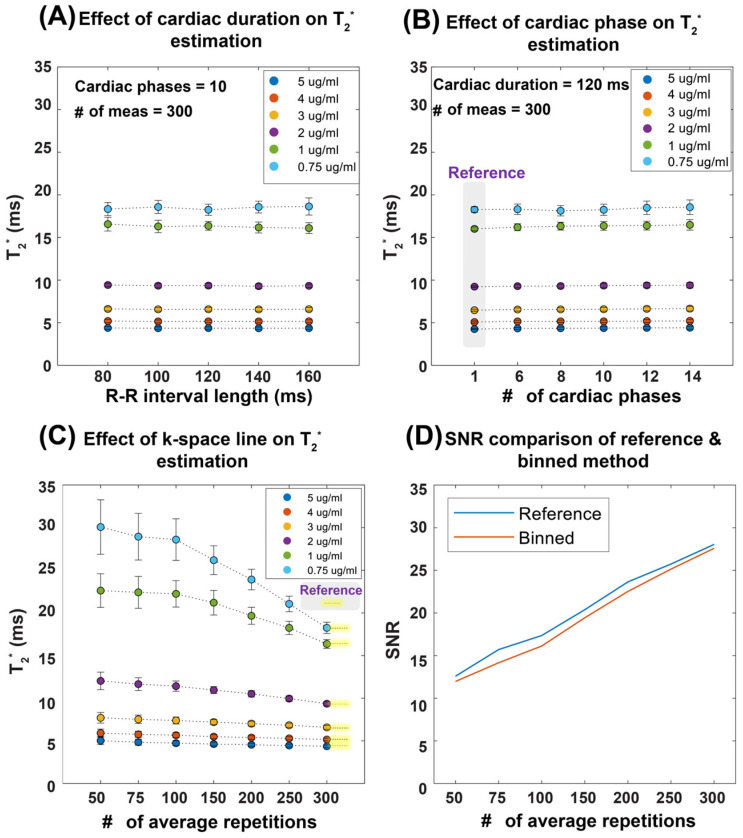
Summary of results obtained from the validation of temporal phase-resolved, retrospectively gated T_2_^*^ mapping in a phantom study. To emulate the in vivo situation, a simulated heart rate was used. The T_2_^*^ mapping performance was evaluated as a function of the heart period, the number of cardiac phases, and the number of repetitions (average). (**A**) Robustness of the proposed binning method against R-R interval length, mimicking the in vivo situation. (**B**) Effect of the number of cardiac phases with a total cardiac phase of one representing a static acquisition without binning, used as the reference. (**C**) The impact of averaging, showing that, as the number of repetitions increases, the T_2_^*^ values converge toward the reference, particularly at 300 repetitions. Averaging is performed in the k-space domain. (**D**) The relationship between SNR and the number of averages for a representative vial with 0.75 µg ferumoxytol per ml of distilled water, illustrating that both the reference and retrospective gating methods exhibit similar SNR trends, though the binning approach results in slightly lower SNR values compared to the reference protocol. Abbreviations: Card Dur: cardiac duration, # of Meas: number of measurements.

**Figure 3:**
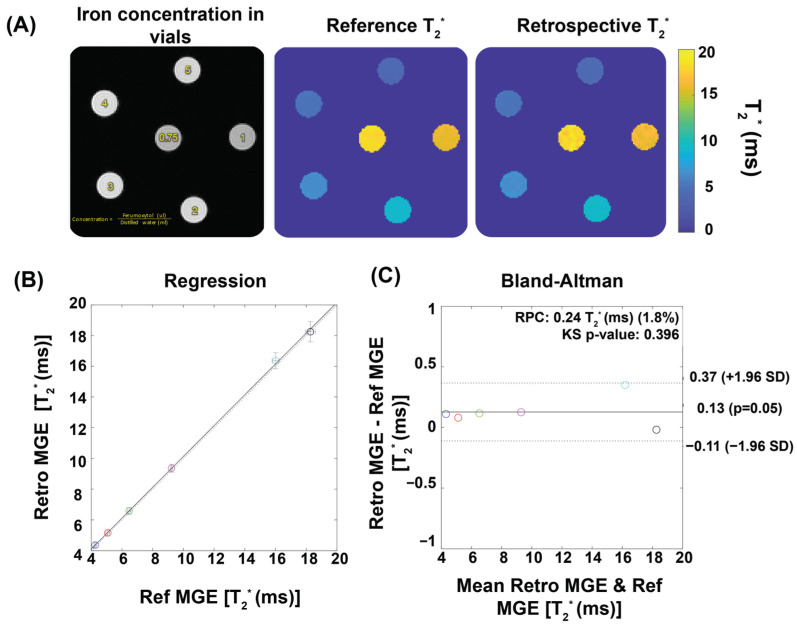
Validation of temporal phase-resolved T_2_^*^ mapping for phantoms using ferumoxytol solutions to mimic myocardial T_2_^*^ properties. (**A**) Qualitative comparison of T_2_^*^ maps. An exemplary first echo image (TE = 1.5) of six iron concentrations reconstructed by the retrospective gating approach together with T_2_^*^ maps of the phantom obtained from reference MGE and from a retrospective gating MGE sorted by a real pulse-oximetry signal recorded from a mouse. (**B**) The retrospective gating approach shows strong correlation with the reference method, as indicated by R^2^ ~ 1. The dotted line shows the identity line, while the solid line illustrates the linear regression fitting. The correlation was assessed using Pearson’s regression. (**C**) The Bland–Altman plot demonstrates a good agreement between the reference and the retrospective gating approach. Each colored circle represents a different ferumoxytol concentration. The dashed lines indicate a confidence level of 95%, while the continuous lines depict the average percentage variances.

Figure 2 demonstrates the performance of our T_2_^*^ mapping approach under different conditions with consistent results obtained for a large range of R-R intervals covering 80–160 ms. The accuracy improved with an increased number of repetitions, while retrospective binning slightly reduced the signal-to-noise ratio (SNR) without altering overall trends (Figure 2A–D). Figure 3 shows T_2_^*^ maps derived from the reference method and the binning approach using real mouse pulse-oximetry signals. The regression and Bland–Altman plots confirm strong agreement between both methods (R^2^ = 1, ICC~1), supporting the robustness of retrospective binning.

### 3.2. Mouse Model Carrying Mybpc3 Gene Mutation Shows HCM Phenotype Revealed by CMR

**Figure 4:**
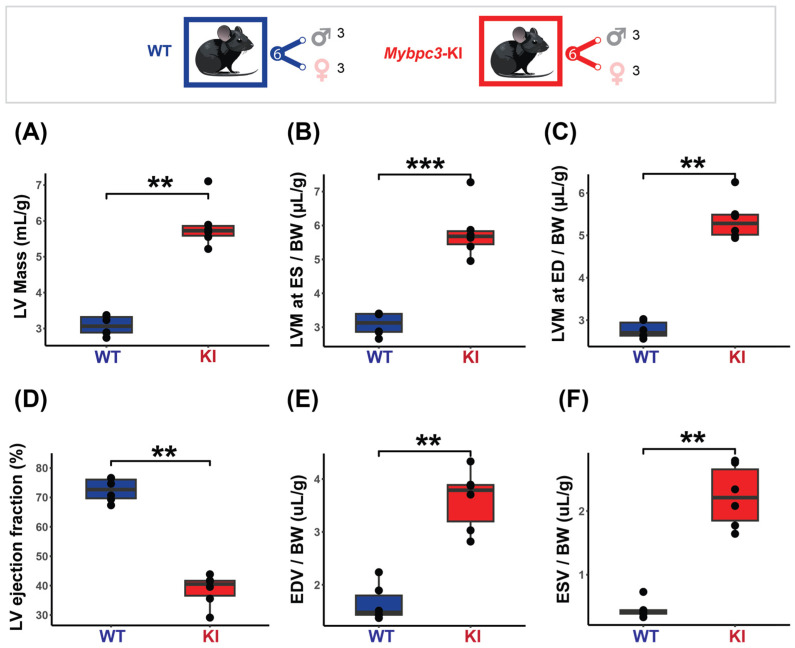
Characterization of the mice cohort using in vivo cardiac MRI shows severe cardiac dysfunction in the HCM (*Mybpc3*-KI) mice. **Top**: Mice cohort included the *Mybpc3*-KI mice (male: *n* = 3, female: *n* = 3) that were compared to the WT mice (male: *n* = 3, female: *n* = 3). (**A**) LV mass normalized to body weight showing significant increase in the *Mybpc3*-KI mice compared to the WT mice. (**B**,**C**) LV mass comparison at both end systole (ES) and end diastole (ED) demonstrates severe hypertrophy in the *Mybpc3*-KI mice compared to the WT mice. (**D**) Cardiac chamber quantification and functional assessments, including left ventricular ejection fraction (LVEF), were conducted on a slice-by-slice basis using manually segmented endo- and epi-cardiac borders in end-systole and end-diastole obtained from short-axis 2D CINE images. LVEF assessment reveals a significant decrease in the *Mybpc3*-KI mice compared to the WT groups. (**E**,**F**) Higher ES and ED volumes in the *Mybpc3*-KI mice indicates reduced cardiac contractility and possible ventricular dilation. All data are presented as a mean ± standard error of the mean. Due to the relatively small sample size in this study, a non-parametric Mann–Whitney U test was used to compare differences in variable distributions between the HCM and the control groups. ** *p* < 0.01, *** *p* < 0.001. Abbreviations: LV: left ventricle; LVM: left ventricular mass; BW: body weight; ED: end diastole; ES: end systole; ESV: end systolic volume; EDV: end diastolic volume; KI: *Mybpc3*-KI; WT: wild type.

The results obtained from the cardiac chamber quantification and the cardiac function assessment in the HCM model are summarized in Figure 4. To ensure comparability and account for size-related differences, all measurements were normalized to body weight. The HCM mice displayed pronounced cardiac hypertrophy, with increased LV mass at ED (KI = 5.37 ± 0.49 µg/g vs. WT = 2.77 ± 0.20 µg/g, *p* < 0.01) and ES (KI = 5.81 ± 0.79 µg/g vs. WT = 3.09 ± 0.33 µg/g, *p* < 0.001) (Figure 4A–C). Regional LV wall thickness was not uniformly increased, with the overall mid and apical regions being significantly thicker in the HCM mice (KI _LVT Mid_ = 50.08 ± 4.68 µm/g vs. WT _LVT Mid_ = 45.80 ± 20.07 µm/g, *p* < 0.01, KI _LVT Apical_ = 51.72 ± 5.76 µm/g vs. WT _LVT Apical_ = 40.50 ± 13.18 µm/g, *p* < 0.01). A significantly lower LVEF was found in the HCM mice (KI = 38.55 ± 5.39% vs. WT = 72.53 ± 3.95%, *p* < 0.01, Figure 4D). The EDV and ESV were also significantly higher in the HCM mice (KI _EDV_ = 3.61 ± 0.57 µL/g vs. WT _EDV_ = 1.65 ± 0.34 µL/g, *p* < 0.01), (KI _ESV_ = 2.23 ± 0.49 µL/g vs. WT _ESV_ = 0.46 ± 0.14 µL/g, *p* < 0.01 (Figure 4E,F). No significant correlation between LVEF and LV mass was found (KI rho = −0.43, *p* = 0.4; WT rho = −0.23, *p* = 0.7).

### 3.3. Cardiac Phase-Resolved T_2_^*^ Mapping Detects Changes Across the Cardiac Phases

**Figure 5:**
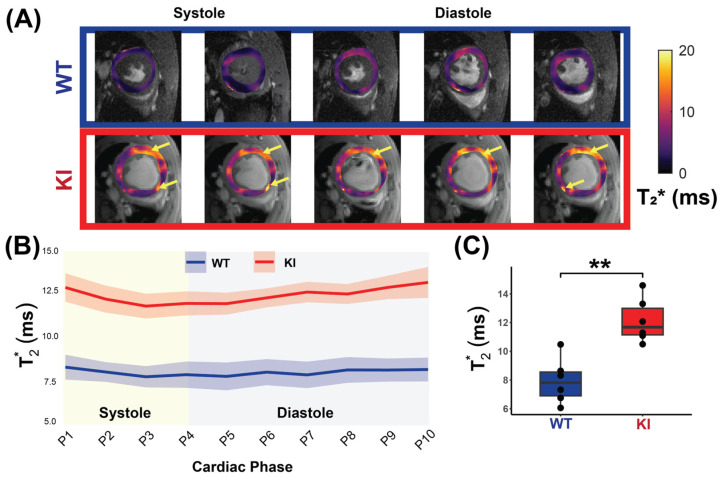
In vivo myocardial T_2_^*^ measurement covering the whole cardiac cycle in WT and in HCM mice. (**A**) Representative T_2_^*^ maps superimposed to anatomic images obtained for 10 cardiac phases for a male WT control mouse (top) and a male *Mybpc3*-KI mouse (bottom) with yellow arrows showing regions of increased T_2_^*^. (**B**) Comparison of T_2_^*^ between the WT controls and the *Mybpc3*-KI HCM mice reveals elevated T_2_^*^ values in the HCM mice across all cardiac phases (P1-P10, *n* = 6 mice for each group). (**C**) Mean myocardial T_2_^*^, averaged over the cardiac cycle for each mouse is significantly higher in the HCM mice compared to controls (*n* = 6, 3 females). Mann–Whitney U test was used to compare differences in variable between the HCM and the control groups. ** *p* < 0.01. Abbreviations: KI: *Mybpc3*-KI; WT: wild type.

Figure 5A shows cardiac phase-resolved myocardial T_2_^*^ maps overlaid on CINE images obtained for the WT and KI mice. Mean myocardial T_2_^*^ values were calculated across the cardiac phases and averaged for each group to examine the T_2_^*^ dynamics across the cardiac cycle (Figure 5B). The HCM mice had significantly higher mid-ventricular myocardial T_2_^*^ values compared to the WT controls (KI _T2*_ = 12.14 ± 1.54 ms vs. WT _T2*_ = 7.93 ± 1.57 ms, *p* = 0.002; Figure 5C).

To investigate T_2_^*^ changes across the cardiac cycle, the Friedman test was conducted. An analysis of all mice, regardless of genotype, revealed significant differences in T_2_^*^ values across cardiac phases (χ^2^ = 23.436, *p* = 0.005). Stratified by genotype, no significant differences were detected in the WT mice (χ^2^ = 5.818, *p* = 0.758), whereas the HCM mice demonstrated significant differences across phases (χ^2^ = 27.818, *p* = 0.001). Comparing T_2_^*^ variability (SD) across genotypes showed no significant difference (*p* = 0.471). These findings suggest a cardiac phase-dependent variability within the KI mice, but no significant difference in overall variability between the WT and the KI mice.

**Figure 6:**
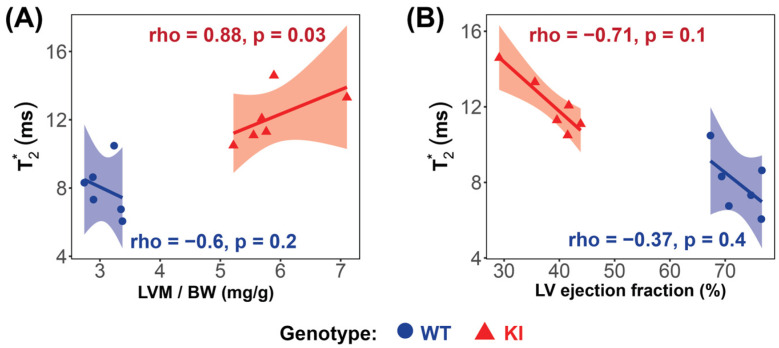
Correlation analysis reveals significant relationships between LV mass and T_2_^*^ in the *Mybpc3*-KI HCM mice. (**A**) A very strong, significant correlation was observed between T_2_^*^ and LV mass in the *Mybpc3*-KI HCM mice (red 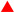), while no significant correlation was found in the *Mybpc3*-WT mice (blue 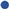). (**B**) No significant correlation was detected between the T_2_^*^ and the LV ejection fraction in either group. Associations were assessed using Spearman’s rank correlation coefficient (ρ), and were considered significant at *p* < 0.05. Abbreviations: LV: left ventricle; KI: *Mybpc3*-KI; WT: wild type.

Significant strong correlations between myocardial T_2_^*^, averaged over all cardiac phases, and LV mass were found in the HCM mice (rho = 0.88, *p* = 0.03; Figure 6A), while no significant correlation was found in the WT group (rho = −0.6, *p* = 0.2; Figure 6A). A strong, though not statistically significant, negative correlation between T_2_^*^ and LVEF was observed in the HCM mice (rho = −0.71, *p* = 0.1) (Figure 6B).

## 4. Discussion

Our exploratory study demonstrates the feasibility of cardiac phase-resolved T_2_^*^ mapping for detecting myocardial changes in HCM using a clinically relevant mouse model. We first validated the retrospective gating scheme used for cardiac phase-resolved image reconstruction on a calibrated static T_2_^*^ phantom and selected the imaging parameters to ensure accurate T_2_^*^ mapping in vivo. Using the retrospectively gated approach, we revealed a significant increase in T_2_^*^ in the *Mybpc3*-KI mice across all cardiac phases.

Elevated T_2_^*^ values have been observed in HCM patients [19], heart failure with preserved ejection fraction (HFpEF) [13], non-ischemic heart failure and dilated hypertrophies [33]. Although we did not find a statistically significant difference in the dynamics of T_2_^*^ changes across the cardiac cycle between the WT control and HCM mouse, our data suggests that T_2_^*^ tends to fluctuate across the cardiac cycle. Additionally, previous clinical findings, where cyclic T_2_^*^ variations were documented in both healthy and HCM subjects, showed septal T_2_^*^ decreasing in diastole and increasing in systole [19,20]. Conversely, studies capturing T_2_^*^ at end-diastole [34], reported a decline in T_2_^*^ in hypertensive patients with LVH, presenting a compelling case for examining the cardiac phase dependent variability of myocardial T_2_^*^.

The pathophysiological significance of our findings likely reflects variations in the blood volume fraction (BVF) between cardiac phases, which influence the amount of deoxygenated hemoglobin per myocardial tissue. In theory, T_2_^*^ is expected to decrease with increased deoxygenated hemoglobin concentration per tissue volume. However, in HCM, the observed elevation in T_2_^*^ suggests a lower deoxygenated hemoglobin effect, which may be linked to a reduced BVF. This reduction may result from microvascular dysfunction, impaired perfusion, and decreased capillary density, all of which can influence myocardial remodeling. Notably, BVF dynamics are closely linked to myocardial contractility—typically assessed through strain measurements—which is often impaired in HCM and correlates with myocardial mass [35]. Supporting this relationship, we observed a significant correlation between T_2_^*^ and LV mass in our HCM mouse model, reinforcing the association between T_2_^*^ changes and myocardial microstructural alterations [13].

Several factors can influence T_2_^*^, including tissue composition [20], myocardial oxygenation [36], diffuse myocardial fibrosis [37], or blood volume fraction [20]. In HCM, complex microvascular remodeling, including capillary rarefaction, altered oxygen extraction, and redistribution of intramyocardial blood volume may contribute to locally increased T_2_^*^, consistent with our observation. Importantly, T_2_^*^ could provide valuable insights into ischemic injury in HCM, overlapping with features seen in chronic coronary syndromes [38]. Furthermore, reduced T_2_^*^ has been associated with ventricular arrhythmias, independent of fibrosis [39,40]. Together, these findings underscore the importance of interpreting myocardial T_2_^*^ in the context of underlying structural and functional microvascular alterations, rather than as a direct surrogate for deoxyhemoglobin content alone.

To better interpret the microstructural changes underlying our findings, it is essential to place our mouse model within the broader spectrum of HCM severity. This mouse model exhibits cardiac-specific expression of *Mybpc3* in the KI mouse, with prior studies confirming markedly reduced mRNA and protein levels in the heart [29,41,42,43]. Consistent with the known phenotype of this model, our *Mybpc3*-KI mice demonstrated early-onset cardiac hypertrophy, typically developing shortly after birth [42,44]. LVEF declines significantly within days, suggesting that our 6–12-week-old mice may represent an intermediate stage of HCM [42,44]. To ensure comparability, normalization was performed using body weight within sex-matched groups, following validated protocols that account for developmental and sex-related variability. Consequently, the elevated T_2_^*^ in our *Mybpc3*-KI mice may reflect early microstructural changes, including cellular hypertrophy, microvascular alterations, and impaired contractility, all of which may precede the development of advanced fibrosis. Further histological analysis is necessary to determine the nature and extent of these changes at this specific age. These would include myocardial fibrosis and capillary density assessments, to complement our imaging data. This will enhance the clinical relevance of our findings and provide a more comprehensive understanding of the myocardial changes associated with the observed T_2_^*^ variations.

To summarize, our findings highlight the potential of cardiac phase-resolved T_2_^*^ mapping to capture early myocardial alterations in HCM, offering insights into disease-related changes that may not be apparent with conventional single phase MRI approaches. While further validation is needed, our cardiac phase resolved approach may serve as a useful tool for investigating dynamic myocardial processes in preclinical models of cardiomyopathy.

### Limitations

While this study demonstrates the utility of full cardiac cycle T_2_^*^ mapping for HCM, several limitations warrant discussion. Our 2D acquisition approach was constrained to a single mid-ventricular short axis view slice of the heart, potentially missing the regional heterogeneity characteristics of HCM, which could be mitigated in future studies through 3D acquisitions with higher spatial resolution.

The discrepancy between our results and those observed in certain human studies regarding T_2_^*^ values may arise from variations in methodology, such as imaging protocols, as well as the differing stages of disease progression in human cohorts compared to our animal model. Additionally, the number of cardiac phases affected the temporal resolution, which was on average around 12 ms, though the increased fat–water shift at higher magnetic fields provided a speed advantage for CINE T_2_^*^ mapping.

This study’s sample size of *Mybpc3* mutation mice was limited, though consistent myocardial changes support the method’s proof-of-concept. The size was guided by a rigorous a priori power analysis, informed by prior studies using similar experimental models [45,46,47].

The absence of histological validation and LGE comparison limits direct correlation with fibrosis; however, our focus was on temporal variations in myocardial T_2_^*^ rather than fibrosis quantification. T_2_^*^ mapping provides a dynamic, non-contrasting agent-based assessment of myocardial function and structure, capturing changes that LGE may overlook. Future research should expand to diverse animal models and human HCM cohorts, while focusing on technical improvements to enhance temporal resolution, spatial coverage, and clinical applicability, ultimately establishing T_2_^*^ mapping as a valuable complement to conventional cardiac imaging for comprehensive HCM assessment.

## 5. Conclusions

This study represents a critical first step in validating human data, demonstrating that cardiac phase-resolved T_2_^*^ mapping using retrospective gating at ultrahigh field MRI is feasible for detecting myocardial alterations in HCM. We observed elevated T_2_^*^ across the cardiac cycle correlating with left ventricular mass in the HCM mice. These findings suggest that phase-resolved T_2_^*^ mapping may offer improved sensitivity for detecting myocardial alterations beyond conventional single-phase approaches, though further validation and complementary molecular and histological assessments are needed to establish clinical relevance.

## Figures and Tables

**Table 1:** Summary of the MRI parameters used in the phantom and in the in vivo studies. The parameters used for multi-echo gradient-echo (MGE) MRI using conventional reconstruction (reference) and retrospective binning MGE are identical.

Study Type	In Vivo	Phantom and In Vivo
Parameters	CINE Imaging	T_2_^*^ mapping
TR (ms)	8.5	14
First TE (ms)	1.58	1.5
Echo Spacing (ms)	---	1.6
Number of Echo Images	---	7
FOV (mm × mm) Slice Thickness (mm) Number of Slices	11 × 22 0.8 16	30 × 30 0.8 10
Matrix Size	192 × 384	128 × 128
Excitation Flip Angle (°)	20°	10°
Receiver Bandwidth (kHz)	98	133
Number of Measurements	---	300
Scan Time		10 m6 s0 ms
Sequence	IntraGate FLASH	MGE–monopolar
Nominal Average ^1^		27
Number of Cardiac Phases	16	10

^1^ The nominal average is determined by the quality of triggering.

## Data Availability

The datasets generated for this study are available from the corresponding author upon reasonable request.

## Abbreviations

The following abbreviations are used in this manuscript:

MRI	Magnetic resonance imaging
CMR	Cardiovascular magnetic resonance
FLASH	Fast low angle shot
TR	Repetition time
TE	Echo time
FOV	Field-of-view
CINE	Cinematic
SAX	Short axis
EF	Ejection fraction
ED	End diastole
ES	End systole
HCM	Hypertrophic cardiomyopathy
LV	Left ventricle/left ventricular
LGE	Late gadolinium enhancement
*Mybpc3*	Cardiac myosin-binding protein c
*MYH7*	β-myosin heavy chain
OMIM	Online Mendelian Inheritance in Man
ECG	Electrocardiogram
SD	Standard deviation
